# SAXS study of the formation and structure of polynuclear thorium(IV) colloids and thorium dioxide nanoparticles

**DOI:** 10.1107/S1600577521012923

**Published:** 2022-01-18

**Authors:** Baihui Zhai, Qiang Tian, Na Li, Minhao Yan, Mark J. Henderson

**Affiliations:** a State Key Laboratory of Environment-Friendly Energy Materials, Southwest University of Science and Technology, Mianyang 621010, People’s Republic of China; b National Facility for Protein Science in Shanghai, Zhangjiang Laboratory, Shanghai 201204, People’s Republic of China

**Keywords:** small-angle X-ray scattering, actinide, ThO_2_, crystallites

## Abstract

Stable actinide colloids and nanoparticles are of interest because of their potential to affect the transportation of radionuclides in the near-field of a nuclear waste repository. At high concentrations, thorium(IV) can precipitate to form intrinsic colloids. In the present study, polynuclear thorium colloids and thorium dioxide crystallites, formed by the condensation of hydrolyzed Th^4+^ solutions (3 m*M*; initial pH 5.5) aged for up to 18 months, were studied using small-angle X-ray scattering. Scattering profiles were fitted using a unified Guinier/power-law model (Beaucage model) to extract the radii of gyration and Porod exponents. Analysis of the scattering profiles from a dispersion aged for 5 months indicated that both polymer coils and more compacted structures (radius of gyration *R*_g_ ≃ 10 nm) were present, which translated in the Kratky plots as a plateau and a peak maximum, respectively. After 18 months, the SAXS data were consistent with the presence of agglomerates of ThO_2_ particles suspended in aqueous solution (pH 3.2; [Th] = 1.45 m*M*). The measured radius of gyration (*R*_g_) of the agglomerates was 5.8 nm, whereas the radius of the ThO_2_ particles was 2.5 nm.

## Abstract 2

The study provides insight into the formation of thorium dioxide nanoparticles in solution using small-angle X-ray scattering.

## 1. Introduction

Thorium, approximately three times more abundant than uranium, has received continuous attention from the nuclear fuel industry owing to its potential to minimize the production of plutonium in reactors based on thorium-based mixed-oxide fuels (Kabach *et al.*, 2020 ▸; Drera *et al.*, 2014 ▸). Current research in actinide (An) nanomaterials includes synthesis of oxide nanoparticles (Shi *et al.*, 2017 ▸) and investigating the effects of size, shape and electronic structure on the physico-chemical properties of the materials. However, applications such as mining extraction, processing, tailings and residues provide a potential pathway for thorium to enter the soil–water environment (Aziman *et al.*, 2021 ▸). Nanoparticles can exhibit much higher toxicities than their bulk forms because of their smaller sizes, larger surfaces areas and greater reactivities. For example, He *et al.* (2019 ▸) studied the toxicity of ThO_2_ nanoparticles on green algal cells. They showed that smaller ThO_2_ particles (≃ 50 nm) had a higher affinity for attachment to the surface of green algal cells than larger particles.

Knowledge of the speciation and chemical behaviors of intrinsic thorium oxide nanoparticles/colloids has potential implications for the behavior of Th(IV) and, by analogy, An(IV) in the environment. Carrier- or pseudo-colloids formed by the adsorption of actinides onto natural aqua­tic colloids such as mineral fragments, humic substances and microorganisms might also increase the mobility of Pu(IV) in the far-field environment of a high-level nuclear waste repository (Romanchuk & Kalmykov, 2020 ▸; Kersting, 2013 ▸; Abdel-Fattah *et al.*, 2013 ▸). Colloids, in the form of amorphous or microcrystalline An(OH)_4_(am) or AnO_2_·*x*H_2_O(s), are solubility-limiting phases that play a key role in transporting the actinides to the near-field environment (Dumpala *et al.*, 2021 ▸; Nishikawa *et al.*, 2018 ▸). For thorium(IV), hydrolysis reactions produce a variety of oxides, hydroxides and complexes. For example, polynuclear species Th_2_(OH)_2_^6+^, Th_4_(OH)_8_^8+^ and Th_4_(OH)_12_^4+^ (Aziman *et al.*, 2021 ▸); ThO_2_ crystallites (Magini *et al.*, 1976 ▸); hydrous thorium dioxide ThO_2_·*n*H_2_O (Dzimitrowicz *et al.*, 1985 ▸); amorpous Th(OH)_4_, aqueous Th(OH)_4_ and crystalline ThO_2_ (Neck & Kim, 2001 ▸); microcrystalline ThO_2_·*x*H_2_O(s), amorphous ThO*_n_* (OH)_4–2*n*_·*x*H_2_O (Rothe *et al.*, 2002 ▸); and [Th_2_(μ_2_-OH)_2_(NO_3_)_6_(H_2_O)_6_]H_2_O, [Th_2_(μ_2_-OH)_2_(NO_3_)_4_(H_2_O)_8_](NO_3_)_2_ and [Th_2_(μ_2_-OH)_2_Cl_2_(H_2_O)_12_]Cl_4_·2H_2_O (Wilson *et al.*, 2007 ▸).

Thorium(IV) is an analog for studying the tetravalent state of the more radiologically and chemically toxic Pu(IV) because of their similar coordination chemistry (Li *et al.*, 2020 ▸) and ionic radii which are 0.108 nm and 0.096 nm for thorium and plutonium, respectively. Despite minor differences in their properties, a tenfold coordination for Th^4^ and a slightly larger ionic radius, recent light scattering measurements of thorium polymers and colloids formed in the acidic solutions tend to highlight the similarities between the hydrolytic reactions of Th(IV) and Pu(IV) (Priyadarshini *et al.*, 2016 ▸). Tetravalent actinide colloids relate to geological disposal of spent nuclear fuel (Zänker & Hennig, 2014 ▸). Among the tetravalent actinide, thorium has attracted interest because of thorium-related contamination of the environment (Aziman *et al.*, 2021 ▸). For example, thorium silicate (Hennig *et al.*, 2013 ▸; Zänker *et al.*, 2016 ▸) and thorite (Estevenon *et al.*, 2020 ▸), which are stable in water at near-neutral pH, have potential implications for colloidal transport in the environment. Speciation diagrams suggest that Th(OH)_4_ or ThO_2_·2H_2_O are major colloids at neutral pH, whereas Th_*x*_(OH)_y_(CO_3_)_2_^(4*x*−*y−2*x**)^ appears in the presence of carbonate, especially at higher pH values (Li *et al.*, 2019 ▸). However, colloidal forms of thorium are reported to mobilize between pH 2 and pH 10 (Li *et al.*, 2019 ▸), the pH range of natural waters (Silva & Nitsche, 1995 ▸).

In this work, small-angle X-ray scattering (SAXS) from polynuclear thorium(IV) colloids and thorium oxide nanoparticles are discussed. SAXS has previously been used to investigate the hydrolysis behavior of Th^4+^ solutions (Magini *et al.*, 1976 ▸). These authors showed that polynuclear thorium complexes form at room temperature, whereas agglomerates of ThO_2_ crystallites are induced by a nonhydro­thermal treatment. Here, we applied the model developed by Beaucage (Beaucage & Schaefer, 1994 ▸; Beaucage, 1995 ▸, 1996 ▸) to SAXS data obtained from thorium polymer/nanoparticle dispersions. Our aim is to highlight the various stable colloidal forms (hydroxide-bridged complexes, oxygen-bridged polymers and oxide nanoparticles) dispersed in aqueous solutions aged for up to 18 months. This knowledge will be used to understand the interaction between Th(IV) colloids and clay buffer material within a proposed deep geological repository.

## 2. Experimental

### 2.1. Materials

Thorium nitrate tetrahydrate (Th(NO_3_)_4_·4H_2_O) was purchased from Chengdu Beyond Chemical Co. Ltd. Sodium hydroxide (NaOH, ≥96.0%, Chengdu Jinshan Chemical Reagent Co. Ltd) was used as received.

### 2.2. Synthesis of thorium colloids and nanoparticles

Thorium colloids were synthesized under a nitro­gen atmosphere to avoid the possible reaction of thorium polymers with carbonates formed in the presence of CO_2_. A stock solution of Th(NO_3_)_4_·4H_2_O (30 ml; 3 mmol l^−1^; pH 2.5), denoted sample T_0_, was adjusted to pH 5.5 (T_1_), 6.0 (T_2_), 6.5 (T_3_), 7.5 (T_4_) and 8.0 (T_5_) using NaOH solution (0.1 mol l^−1^). Two sets of mixtures were made. An aliquot of the clear colorless dispersion was removed above the precipitate when synchrotron SAXS beam time became available, *i.e.* after 26 days and 166 days.

### 2.3. Characterization methods

The synchrotron SAXS measurements were performed at the BL19U2 beamline at the Shanghai Synchrotron Radiation Facility (China). A flow cell made of a quartz capillary with a diameter of 1.5 mm and a wall thickness of 10 µm was used to withdraw an aliquot of thorium dispersions. The SAXS patterns were recorded using a Pilatus 1M detector (DECTRIS) with a pixel size of 172 µm. Intensity profiles were denoted *I*(*Q*) and plotted as the magnitude of the scattering vector magnitude *Q* = (4πsinθ)/λ, where λ is the wavelength of the incident X-rays and θ is half the scattering angle. The wavelength was tuned to 0.09184 nm and the sample-to-detector distance was 5660 mm, resulting in the *Q* range 0.07–3.6 nm^−1^.

The SAXS measurements were also performed with a SAXSpace instrument (Anton Paar, Austria; 40 kV and 50 mA) when conformational changes were no longer observed. The wavelength of the X-ray radiation was λ = 0.1542 nm. The SAXS patterns were recorded using a Mythen2 R 1K (Dectris, Switzerland) detector, positioned 317 mm from the samples. The recorded patterns showed the *Q* range 0.05 nm^−1^−7.8 nm^−1^, which has an extended *Q* range towards wide angles compared with the setup of the BL19U2 beamline and, as a result, the SAXSpace instrument has an advantage for measuring small particles. The data were fitted by the least-squares method using the *SASfit* software (version 0.94.6; Breßler *et al.*, 2015 ▸) according to the unified Guinier/power-law (Beaucage & Schaefer, 1994 ▸; Beaucage, 1995 ▸, 1996 ▸),

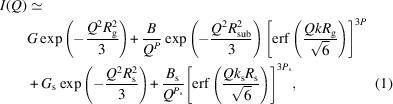

where *R*_g_, *R*_sub_ and *R*_s_ are the radius of gyration of large, medium and small structures in the studied system, respectively; *P* and *P*_s_ are the scaling exponents of the power-law scattering from the large and small structures; and *G* and *B* are the Guinier and the Porod law prefactors.

Transmission electron microscopy (TEM) was performed using a Zeiss Libra200FE microscope by droplet evaporation of the Th dispersion on a Cu TEM grid. Solution pH measurements were recorded with an Accumet XL200 pH (Fischer Scientific) meter equipped with an E-201F pH probe (Shanghai REX Sensor technology Company). Thorium levels were determined by Inductively Coupled Plasma Optical Emission Spectrometry (ICP-OES) analyses using an ICP-OES 730 instrument (Agilent).

## 3. Results

**Figure 1:**
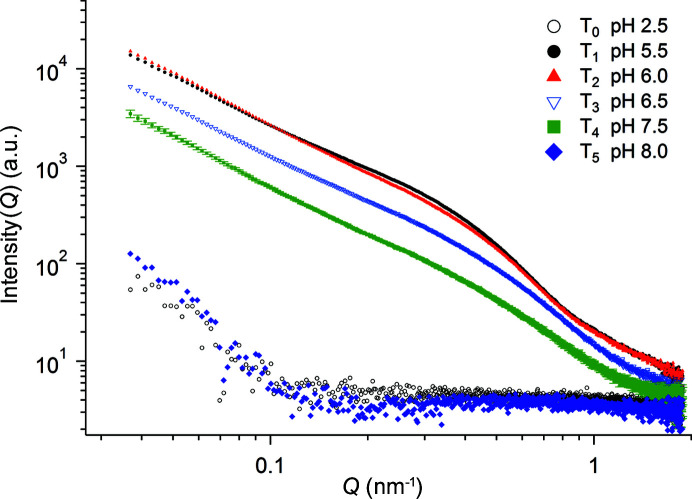
Synchrotron radiation SAXS profiles obtained from aqueous thorium solutions prepared at initial pH values 2.5–8.0 and aged for 166 days: T_0_ (open circles), T_1_ (filled black circles), T_2_ (filled red triangles), T_3_ (open blue triangles), T_4_ (filled green squares) and T_5_ (filled purple diamonds).

The SAXS profiles of samples T_0_–T_5_ prepared between pH 2.5 and pH 8.0 and aged for 166 days are shown in Fig. 1 ▸. Scattering intensities were found to decrease with an increase in the initial pH. At the highest pH limit the scattering profile was found to be similar to that of the thorium stock solution, T_0_, from which all the dispersions were made. Note that with the exception of sample T_0_, the pH values of all solutions decreased from their initial values to pH 3.5 (T_1_), pH 3.4 (T_2_), pH 3.7 (T_3_), pH 3.7 (T_4_) and pH 4.3 (T_5_) after 166 days. Also, only samples prepared under the most acidic conditions at pH 5.5 showed scattering after they were aged for 26 days (not reported).

**Figure 2:**
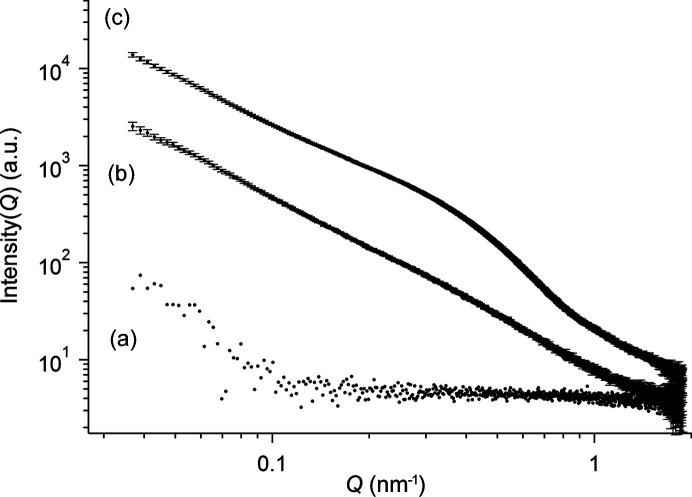
Synchrotron radiation SAXS profiles obtained from (*a*) sample T_0_, (*b*) sample T_1_ at 26 days (pH 4.1) and (*c*) sample T_1_ at 166 days (pH 3.5).

These results suggest that both the solution acidity and aging time direct the amount of thorium colloids in solution above the precipitate. Based on these preliminary results, the scattering profiles from sample T_1_ aged for 26 days and for 166 days were selected for further analysis. The synchrotron-radiation SAXS profiles for the Th^4+^ stock solution and the sample T_1_ aged for 26 and 166 days are shown in Figs. 2 ▸(*a*)–2(*c*). The SAXS profile of the dispersion after 26 days [Fig. 2 ▸(*b*)] showed strong scattering and a power scattering (*I* ∝ *Q* ^−α^ with exponent α = 1.7) in the *Q* range from 0.04 nm^−1^ to 0.2 nm^−1^, which is characteristic of scattering from a self-avoiding random walk chain (Schurtenberger, 2002 ▸). The features of this scattering profile and a concomitant fall in the solution pH from 5.5 to 4.1 were interpreted in terms of the presence of hydroxyl-bridged structures, namely, Th_*x*_(OH)_*y*_^(4*x*−*y*)^ polymers (Wickleder *et al.*, 2006 ▸). The scattering profile obtained from the dispersion aged for 166 days [Fig. 2 ▸(*c*)] shows an additional feature at *Q* ≃ 0.35 nm^−1^, an indication that the thorium polymers coexist with smaller objects in the dispersion.

**Figure 3:**
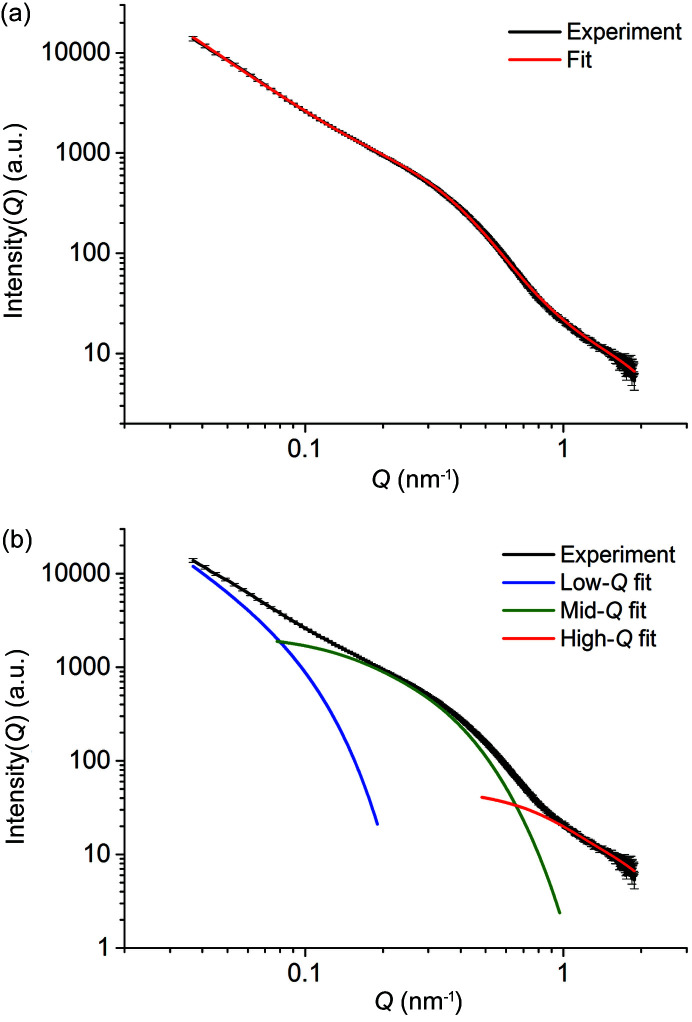
(*a*) Experimental data (closed black circles) for SAXS from sample T_1_ (pH 3.5) aged for 166 days. The red line is the approximation by the unified Guinier/power-law equation. (*b*) Colored lines are the fitting functions obtained using the unified Guinier/power-law equation for the low-*Q* (blue line), mid-*Q* (green line) and high-*Q* (red line) ranges.

The scattering profile shown in Fig. 2 ▸(*c*) was analyzed by the unified Guinier/power-law (Fig. 3 ▸). The SAXS curve displays power slopes at low-*Q* (0.03–0.24 nm^−1^), mid-*Q* (0.2–0.8 nm^−1^) and high-*Q* (0.5–1.9 nm^−1^) ranges, *i.e.* three structural levels are described. At low-*Q* the power-law exponent (1.7) was found to be similar to that obtained from the profile after 26 days. Note that because the size of the polymer network was beyond the maximum size limit of this instrument (π/0.03 ≃ 100 nm), *R*_g_ was arbitrarily set to a higher value, in this case 200 nm. At mid-*Q* the fit yielded a power-law exponent of 2.3, which relates to a branched fractal network more compacted than that displayed by the polymers. The radii of these new objects were estimated to be ∼10 nm. At high-*Q*, a power-law regime with an exponent of 4 is observed, and *R*_g_ ≃ 1.8 nm corresponds to the gyration radius of smallest particles found in this sample. The results of the analysis are summarized in Table 1 ▸.

**Figure 4:**
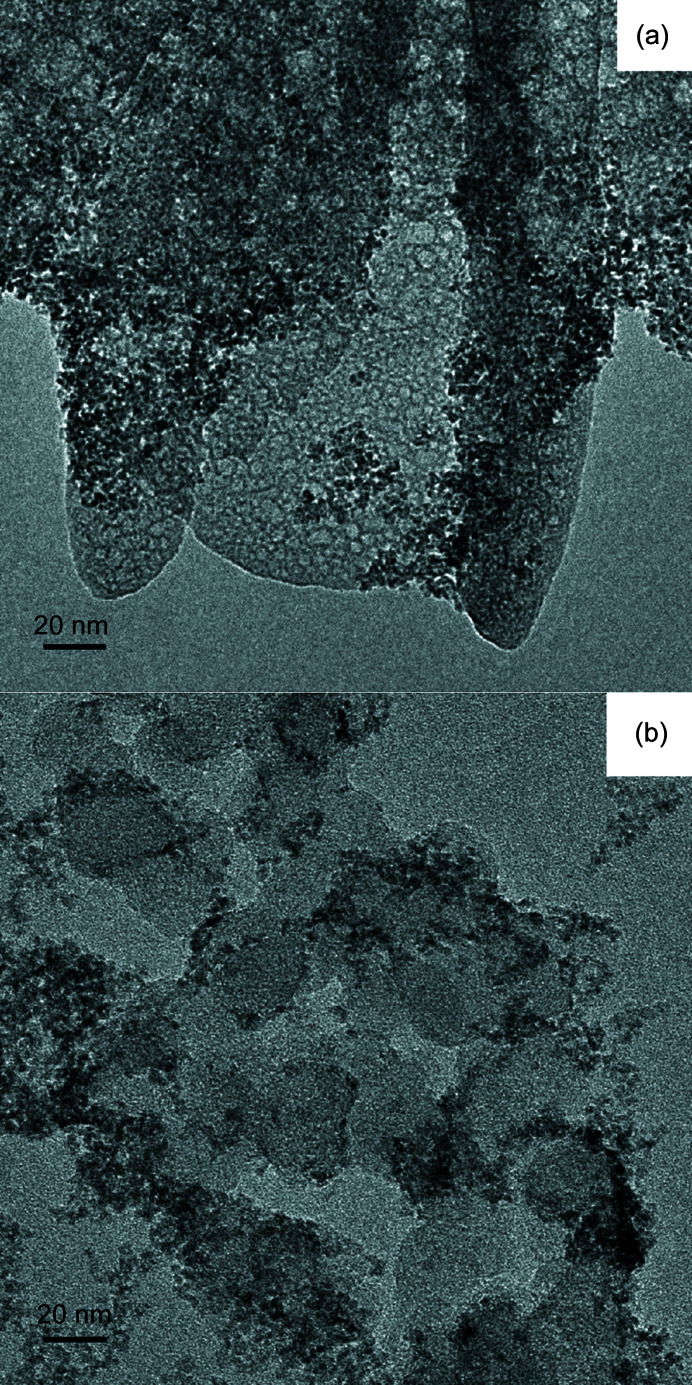
Transmission electron micrographs obtained from aqueous thorium dispersions aged for approximately 6 months: (*a*) T_1_ and (*b*) T_3_.

Transmission electron micrographs obtained from samples T_1_ and T_3_ after aging for 6 months (Fig. 4 ▸) show two features. One is the high-density particles with radii < 2 nm, in agreement with the Beaucage modeling of the high-*Q* SAXS data. The other is the low-density network, which can be attributed to dried Th(IV) polymer solution species, as indicated by the power-law exponents of 1.7 and 2.3 derived from SAXS data (Table 1 ▸).

**Figure 5:**
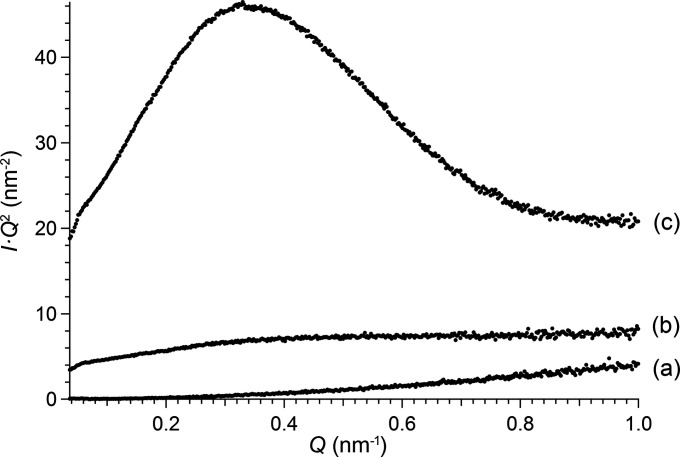
Kratky plots (*IQ* ^2^–*Q*) obtained from SAXS measurements: (*a*) sample T_0_, (*b*) sample T_1_ at 26 days (pH 4.1) and (*c*) sample T_1_ at 166 days (pH 3.5). Data from Fig. 2 ▸.

To highlight the polymer and dense particle structures, the SAXS data of Fig. 2 ▸ were expressed as Kratky plots (*IQ* ^2^–*Q*) (Fig. 5 ▸), which are useful to highlight changes in the compactness of polymers (Fairclough *et al.*, 1999 ▸) and proteins (Kikhney & Svergun, 2015 ▸) in solution. A bell-shaped curve is obtained when the scattered intensity decays as *Q* ^−4^, *i.e.* scattering from solid objects. A plateau at high-*Q* values is obtained when the scattering intensity decays as *Q* ^−2^, *i.e.* scattering from extended structures such as a Gaussian chain. For the thorium dispersions used here, the transition from polymers to oxide particles was characterized by a plateau [Fig. 5 ▸(*b*)] and a bell-shaped curve [Fig. 5 ▸(*c*)] indicating a coil and a compacted structure, respectively.

**Figure 6:**
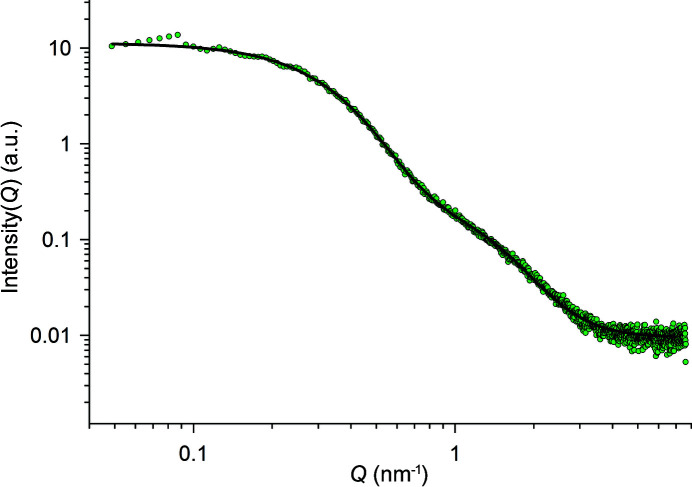
SAXS profile (Anton Paar SAXSpace) obtained from a thorium dispersion aged for 18 months, pH 3.2 (green circles), [Th] = 1.45 m*M*. The unified exponential-power-law fit to the data is shown as a black line.

Prolonged aging of the dispersion for 18 months resulted in a gradual decrease of solution pH from 3.5 to 3.2. SAXS measurements of this dispersion were recorded (Fig. 6 ▸). The *I*–*Q* curve shows a plateau at *Q* < 0.2 nm^−1^, a strong decay at 0.2 nm^−1^ < *Q* < 0.8 nm^−1^, a knee at 0.8 nm^−1^ < *Q* < 2 nm^−1^ and a background at *Q* > 4 nm^−1^. This result implies that two levels of particle structure were present in the dispersion.

The size and shape of the scattering objects were extracted from the measured data using a unified exponential-power-law model using two structural levels. The results of the Beaucage model analysis showed that two particle sizes of radii (*R*_g_) 5.8 nm and 2.5 nm were present in this dispersion (Table 2 ▸).

**Figure 7:**
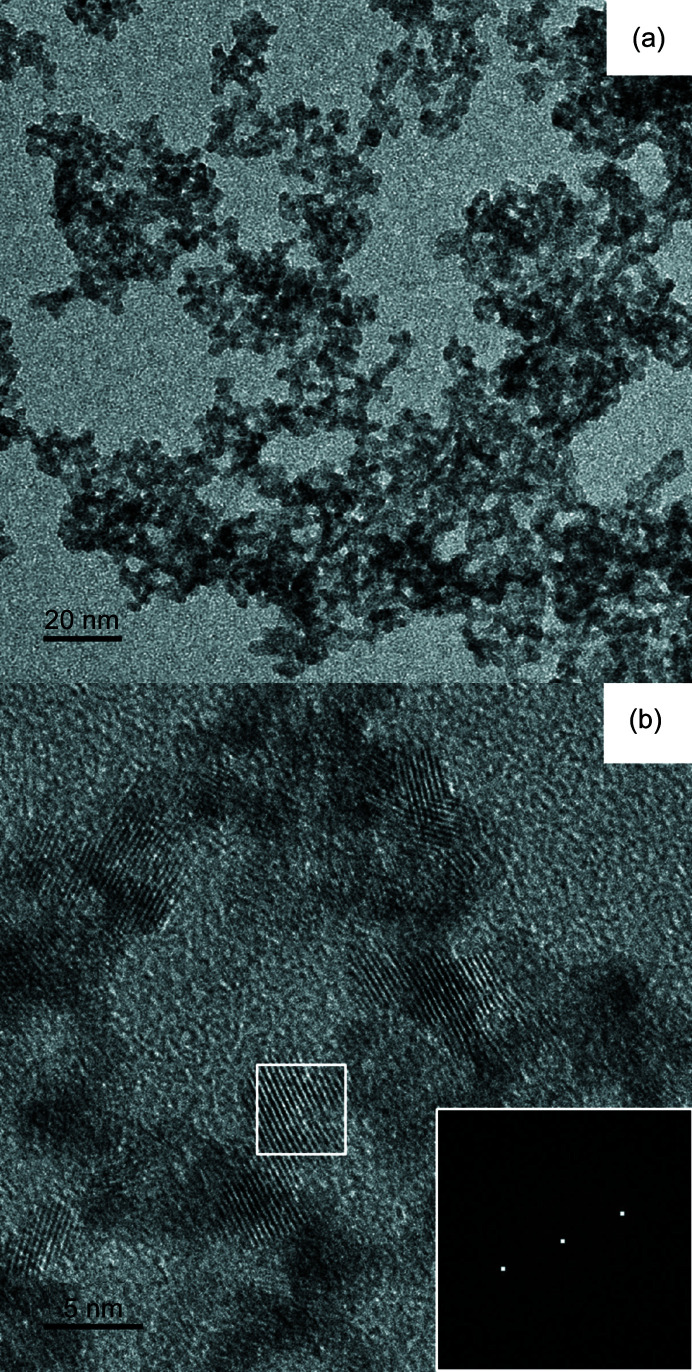
Transmission electron micrograph results from ThO_2_ particles obtained from an aqueous dispersion prepared by hydrolysis of Th(NO_3_)_4_·4H_2_O solution and aged for 18 months: (*a*) low-resolution image, (*b*) high-resolution image showing the lattice fringes of a crystallite and its corresponding FFT (inset).

A TEM micrograph of the particles obtained from the aged dispersion is shown in Fig. 7 ▸(*a*). The agglomerations are composed of monodisperse primary particles of approximately 5 nm diameter [Fig. 7 ▸(*b*)] in agreement with the SAXS Beaucage modeling.

The size was also similar to the mean diameter found for ThO_2_ nanoparticles synthesized by a non-aqueous route (Hudry *et al.*, 2013 ▸). Furthermore, the lattice fringes shown in Fig. 7 ▸(*b*) indicate the microcrystalline structure of the primary particles [Fig. 7 ▸(*b*)]. This lattice space (0.34 ± 0.02 nm) is consistent with the value obtained from a precipitate with the composition ThO_2_·2.5H_2_O(s) (0.34 nm) (Dzimitrowicz *et al.*, 1985 ▸) despite the different method of sample preparation, namely, fast precipitation from concentrated thorium solution at near-neutral conditions. More recently it was shown that freshly precipitated thorium prepared by titration using base consists of a hydrated oxyhydroxide, ThO_*n*_(OH)_4–2*n*_·*x*H_2_O(am) (Rothe *et al.*, 2002 ▸). In the present study, SAXS and TEM results led to the conclusion that agglomerates of microcrystalline ThO_2_ particles (5 nm) were formed in solution after 18 months. The results are in agreement with the formation of agglomerates of thorium dioxide crystallites (3–4 nm) induced by thermal treatment of an aqueous solution of hydrolyzed thorium species (Magini *et al.*, 1976 ▸).

## 4. Discussion

Our interest is the formation of thorium polymers and particles in solution above the precipitate after the initial hydrolysis step, *e.g.* polynuclear hydroxide complexes Th_*x*_(OH)_*y*_^4*x*−*y*^ and thorium(IV) colloids [Th(OH)_4_(am)] (Rothe *et al.*, 2002 ▸). Our SAXS study builds on the work by Magini *et al.* (1976 ▸) who studied the speciation of hydrolyzed thorium solutions at room temperature and at 80–90°C by small-angle and wide-angle X-ray scattering. The solution hydrolyzed at room temperature contained the suggested complex [Th_6_O(OH)_6_(H_2_O)*_n_*]^16+^, whereas the heat-treated solutions contained agglomerates of ThO_2_ particles (Table 3 ▸).

In this study we have applied the unified Guinier/power-law model to SAXS data obtained from thorium polymer/nanoparticle dispersions above the precipitate after aging the sample for 5 months and 18 months at room temperature. The model was particularly useful when applied to the SAXS profile obtained from a dispersion aged for 5 months where the Guinier region, which defined the radius of gyration (*R*_g_ = 10 nm) of the colloids, was obscured by low-*Q* scattering from the thorium polymers. Based on the power-law exponent of 2.3 extracted from the Beaucage modeling of the mid-*Q* range SAXS data, which indicates a loose-packed structure, these particles are most likely amorphous hydroxide Th(OH)_4_(am) or hydrous oxides ThO_2_.*x*H_2_O(am). Furthermore, the differences in these thorium colloid structures were translated in the Kratky plots as either a plateau, a characteristic feature of an ideal Gaussian chain, or a peak maximum suggesting scattering from a more condensed phase. After 18 months, the sizes of two discrete populations of primary (*R*_g_ = 2.5 nm) and secondary particles (*R*_g_ = 5.8 nm) could be quantified by application of the Beaucage model to the scattering curve. In short, the SAXS technique was able to discern polymer from particles, and in the fully aged dispersion discern two discrete populations of primary and secondary particles.

**Figure 8:**
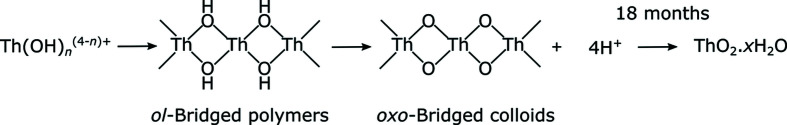
Summary of the hydrolytic behavior of Th^4+^solution (3 m*M*) from an initial pH 5.5, and aged for 18 months at room temperature (pH 3.2; [Th] = 1.45 m*M*).

The SAXS profile (Fig. 6 ▸) shows a plateau at *Q* < 0.2 nm^−1^, an indication that Th_*x*_(OH)_*y*_^(4*x*−*y*)^ polymers were not present in solution after prolonged aging at room temperature. The pH of this dispersion was 3.2. This result is consistent with the formation of the oxo-species by the loss of a proton from each hydroxyl group rather than by the irreversible elimination of water (Johnson & Toth, 1978 ▸; Dumpala *et al.*, 2021 ▸). Based on the results from this study, the hydrolytic behavior is summarized in Fig. 8 ▸.

These ThO_2_ nanoparticles are designated as microcrystalline ThO_2_, *i.e.* most likely they have a hydrated surface and correspond to the generalized composition ThO_2_·*x*H_2_O(s) (Rothe *et al.*, 2002 ▸). In this report, the Beaucage model described both Guinier and Porod regions of a scattering curve, and was used to obtain the radii of gyration and power scattering exponents from different structural levels. More recently it was shown that unwanted artifacts are introduced when the Guinier and Porod prefactors are allowed to vary independently (Hammouda, 2010 ▸). However, the scattering curves shown in Figs. 1 ▸ and 2 ▸(*c*) were sufficiently featured, *e.g.* by a prominent knee at mid-*Q* values to warrant the use of the Beaucage model.

## 5. Conclusions

Thorium colloids dispersed in aqueous solution and aged for up to 18 months were studied by SAXS. The results of the Beaucage modeling demonstrated that the dispersion aged for 5 months contained polynuclear Th(IV) solution species including colloids ∼20 nm in diameter, and thorium dioxide particles (3−4 nm). These SAXS results suggest that the polymers have a low-density network. However, the dispersion aged for 18 months contained only microcrystalline ThO_2_ particles (5 nm) agglomerated to form larger particles (∼12 nm). Further work is necessary to examine the generation and stability of thorium nanoparticles under near-neutral pH conditions.

## Figures and Tables

**Table 1:** Parameters obtained from the fitted SAXS profile obtained from an aqueous thorium solution aged for 166 days (sample T_1_; pH 3.5)

Low-*Q*		Mid-*Q*		High-*Q*	
*R*_g_ (nm)	*P*	*R*_s_ (nm)	*P*	*R*_s_ (nm)	*P*_s_
200	1.7 ± 0.1	9.5 ± 0.2	2.3 ± 0.1	1.77 ± 0.07	4.0

**Table 2:** Parameters extracted from the fitted SAXS profile obtained from an aqueous thorium solution aged for 18 months (sample T_1_; pH 3.2)

*R*_g_ (nm)	*P*	*R*_s_ (nm)
5.8 ± 0.5	4	2.5 ± 0.5

**Table 3:** Sizes of intrinsic thorium colloids suspended in aqueous solution

Colloid designation	pH	Size (nm)	Technique	Reference
ThO_2_ crystallites	–	4 and 17	SAXS	Magini *et al.* (1976 ▸)
Microcrystalline ThO_2_	1.5–2.6	16–23	LIBD†	Bundschuh *et al.* (2000 ▸)
Th(OH)_4_(am)	3.62	>5	LIBD	Rothe *et al.* (2002 ▸)
Cationic hydrous ThO_2_	2.0–2.5	1–1.7	TEM	Lünsdorf *et al.* (2006 ▸)
Thorium colloids	2.9–3.3	13.4–19.6	LIBD	Dumpala *et al.* (2021 ▸)
Microcrystalline ThO_2_ ‡	3.2	5 and 12	SAXS	This study

† Laser-induced breakdown detection.

‡ Dispersion aged for 18 months.
